# Experimental Models for Assaying Microvascular Endothelial Cell Pathophysiology in Stroke

**DOI:** 10.3390/molecules15129104

**Published:** 2010-12-10

**Authors:** Susanna Camós, Judith Mallolas

**Affiliations:** Department of Neurology, Girona Biomedical Research Institute (IdiBGi), Dr. Josep Trueta University Hospital, Av de França s/n, 17007 Girona, Spain; E-Mail: scamos.girona.ics@gencat.cat (S.C.)

**Keywords:** neuroprotection, blood brain barrier, brain endothelial cells, cerebral ischaemia, angiogenesis

## Abstract

It is important to understand the molecular mechanisms underlying neuron death following stroke in order to develop effective neuroprotective strategies. Since studies on human stroke are extremely limited due to the difficulty in collecting post-mortem tissue at different time points after the onset of stroke, brain ischaemia research focuses on information derived from *in-vitro* models of neuronal death through ischaemic injury [1]. This review aims to provide an update on the different *in-vitro* stroke models with brain microvascular endothelial cells that are currently being used. These models provide a physiologically relevant tool to screen potential neuroprotective drugs in stroke and to study the molecular mechanisms involved in brain ischaemia.

## 1. Introduction

### 1.1. Neuroprotection in Cerebral Ischaemia

Neurodegenerative disorders and chronic disability due to stroke affect a large sector of the population. The identification of multiple molecular mechanisms and mediators of ischaemic brain injury constituting potential targets of neuroprotection and the development of neuroprotective drugs and therapies are two of the main challenges in stroke research [2,3]. Although neuroprotection for ischaemic stroke has been a subject of intense research for more than half a century, no therapeutic applications have yet been found [4]. Furthermore, many clinical trials of potential agents have been unsuccessful as the pre-clinical case was not sufficiently well established. To improve this situation, optimal *in-vitro* ischaemic models are needed to study the molecular mechanisms and to test potential neuroprotective drugs prior to considering whether clinical trials are appropriate. To this end, rigorously conducted *in-vitro* studies consisting of oxygen and glucose deprivation assays in cell culture (in an attempt to mimic pathophysiological stroke conditions) are extremely important to provide a reliable, technically straightforward and physiologically relevant model, useful for the study and screening of potential therapeutic targets of neuroprotection against stroke [5]. This review summarizes current knowledge of *in-vitro* ischaemic models with brain microvascular endothelial cells (BMEC) used to study the molecular mechanisms involved in brain ischaemia as well as to assay potential therapeutic strategies with neuroprotective drugs.

### 1.2. Angiogenesis and Neuroprotection

Angiogenesis, the physiological process involving the growth of new blood vessels from pre-existing vessels, is essential in physiological processes and pathological conditions such as diabetic retinopathy, psoriasis, tumour growth and metastases, and stroke [5]. Folkman was a pioneer in emphasizing the importance of understanding the mechanism of the angiogenic process at the biochemical and molecular levels given the potential diagnostic and therapeutic benefits in a wide range of diseases [6], particularly in tumour angiogenesis [7,8]. The angiogenic process is characterized by a combination of the sprouting of new vessels from the sides and ends of pre-existing ones or by longitudinal division of existing vessels with periendothelial cells (intussusceptions), either of which may then split and branch into precapillary arterioles and capillaries. In order for the process of angiogenesis to proceed properly during physiological and pathological conditions, it is essential that a complex array of angiogenic and anti-angiogenic factors, interacting with multiple cells and tissues, be tightly regulated. Moreover, although endothelial cells are the main cell type in the vascular unit, they cannot complete the angiogenic process alone as they need periendothelial cells and matrix components, which provide molecular and functional support.

Angiogenesis involves a variety of coordinated events, including degradation of the extracellular matrix surrounding the parent vessel, migration and proliferation of the endothelial and mural cells to assemble the new vessel, lumen formation, and construction of the mural cell layer of the vessel wall with associated pericytes and/or smooth muscle cells [9]. In Table 1 we summarize the process that occurs in each step of angiogenesis together with the participating molecules.

A challenge for current research into neuroprotection in stroke is to focus on the angiogenic process [10]. Cerebral endothelial cells perform essential functions including maintenance of the blood brain barrier (BBB) and regulation of vascular tone by release of vasoactive factors. In addition, the endothelium plays a key role in injury responses such as inflammation, angiogenesis, and the release of trophic factors [11,12]. Therefore, the proper function and activity of the brain microvasculature network, specifically of the endothelial cells before and during the cerebral ischaemia is extremely important as it may provide a better response to ischaemic brain injury due to blood flow alterations, performing functions such as biochemical support and maintenance of extracellular ion balance. For this reason, many studies that aim to study the effect of different neuroprotective agents on neuronal survival in cerebral ischaemia use (BMEC) cultures to perform their experiments.

Protection of the structural and functional integrity of brain microvasculature may, therefore, improve the cerebral ischaemia outcome. Some authors have focused on ischaemic preconditioning (PC) effects in cerebral endothelial cells [13,14,15]. Ischaemic PC was first described in the heart [16]. In heart tissue, repeated short periods of ischaemia have been found to protect against subsequent longer periods of ischaemia with reperfusion [14]. Subsequent studies have shown that the brain can also be preconditioned to reduce ischaemic brain damage. Brain ischaemia tolerance has been demonstrated both in *in-vivo* [17] and *in-vitro* neuronal cells culture in many studies [13,18,19,20,21]. It has been demonstrated that *in-vivo* ischemic PC reduces BBB disruption and brain oedema formation following focal cerebral ischaemia in rat. Moreover, it has also been associated with less induction of heat shock protein 70, a stress marker, in the cerebrovasculature [22,23]. 

In order to exploit angiogenesis therapeutically in stroke, it is essential that we understand the precise molecular mechanisms involved [10]. This is especially important as some growth factors in post-ischaemic angiogenesis may increase vascular permeability resulting in haemorrhagic transformation [22,24,25]. The aim, therefore, must be to accelerate cerebral angiogenesis without exacerbating brain oedema in stroke [26].

#### 1.2.1. *In-vitro* angiogenesis assays

Different *in-vitro* methods are used to study the effects of a particular treatment or test substance on the angiogenic process. The principal *in-vitro* assays include those for matrix degradation (matrix invasion assays), proliferation (MTT; Thimidine incorporation; BrdU incorporation, cell cycle analysis), apoptosis (TUNEL assay; Annexin V assay), migration (Transwell/Boyden chamber assay; phagokinetic track; wound healing; under agarose assay), and differentiation or tubule formation (matrix assays: Matrigel, collagen/Matrigel, collagen sandwich; 3D gel; co-culture) of endothelial cells, and stem cell organ culture assays (embryoid body; aortic ring assay: rat aortic ring, chick aortic arch, porcine carotid artery; placental vein disk; fetal bone explants: mouse metatarsal assay). Full descriptions of the different specific assays as well as an account of their advantages and limitations can be found in the following reviews [5,27,28].

The assays used for the study of endothelial cell function have the advantages of providing a high level of reproducibility, precision, and sensitivity. Different parameters, such as the spatial and temporal concentration of angiogenic mediators, can be controlled and individual steps in the angiogenic process can be studied separately. These assays have the further advantage of being relatively inexpensive. The most important limitations are that they are technically difficult to set up and comparison between different studies is difficult due to the diversity of reagents employed (endothelial cell origin and passage number, content of Matrigel substrate, growth medium).

Whole or partial organ culture assays are considered to be those which come closest to mimicking the *in-vivo* situation given that they include the surrounding non-endothelial cells (smooth muscle cells and pericytes) as well as a supporting matrix. These explants assays are more representative of the situation found *in vivo* where angiogenesis is triggered and quiescent endothelial cells respond by becoming proliferative, migrating out from the existing vessels, and differentiating into tubules. However, there are still important limitations. Amongst these are the fact that non-human tissues are used and that these models use large vessels whereas angiogenesis is a microvascular event. Moreover, the assays using explant tissues from growing embryos are not truly representative of the stimulation of non-proliferative endothelial cells since endothelial cells undergo proliferation before explantation. In Table 2, we summarize the advantages and limitations of the most important *in-vitro* angiogenesis assays used with endothelial cells and organ culture.

To fully understand the effects of a particular test substance on the process of angiogenesis, more than one *in-vitro* assay must be used to investigate the different steps in the pathway using endothelial cells from different sources. These should be followed with more than one *in-vivo* assay to ensure that *in-vitro* and *in-vivo* results correlate.

## 2. *In-Vitro* Cerebral Ischaemic Models with Microvascular Endothelial Cells

Oxygen-glucose deprivation (OGD) experiments aim to study a variety of pathologies involving ischaemic processes. They consist of an *in-vitro* system that mimics several important aspects of ischaemia, including low oxygen pressure, low nutrient levels (glucose), and the accumulation of cellular products thought to contribute to damage during ischaemia [29]. Given this, these experiments are used to mimic the physiopathological conditions in stroke, in order to study the response of BMECs to ischaemia and to evaluate how changes in their function might affect neuronal survival and recovery after ischaemia. The OGD experimental model consists of replacing the conventional culture medium by a buffer solution or another medium without glucose (sometimes deoxygenated by bubbling with N_2_) and transferring the cells (cerebral endothelial cells, in this case) to a temperature controlled hypoxia chamber (37 °C ± 1) connected to a constant N_2_ flux maintained in a humidified atmosphere. A reperfusion protocol is then performed which consists of replacing the OGD medium with a glucose-containing medium (conventional medium or OGD medium supplemented with glucose) and then transferring the cells to a normoxic incubator to mimic the establishment of the brain blood flow and post-stroke reoxygenation.

This *in-vitro* system was initially used in 1988 by Hlatky *et al.* in their oncological studies to mimic necrosis conditions [30]. In 1998 Berger *et al.* used cortex slices and submitted them to OGD conditions in order to perform ischaemic studies [31]. Over the last ten years OGD has been widely used as an *in-vitro* stroke model with a variety of cellular types (in a single or co-culture system) from different species in numerous ischaemia-related studies.

### 2.1. Cellular Model

The endothelium presents phenotypic heterogeneity and differential features depending on the context of the individual vascular beds or organs. Endothelial cells in arteries are long and narrow and are aligned in the direction of undisturbed flow. The arterial endothelium is continuous with many tight junctions and is characterized by the expression of specific markers including Ephrin B2, DII4 (delta-like 4), ALK1 (activin-receptor-like kinase), EPAS-1 (endothelial PAS domain protein 1), Hey1/2, Depp (decidual protein induced by progesterone), and NRP1 (neutropilin 1). The vein endothelium is also continuous but the cells are shorter and wider and are not aligned in the direction of the blood flow and they possess valves. Specific markers include EphB4, NRP2, and COUP-TFII. The post-capillary venule endothelium is characterized by having thin areas of caveolae and thick portions of VVOs (vesiculo-vacuolar organelles) as well as disorganized tight junctions, reflecting the role of this blood vessel type in mediating inflammation-induced extravasation of leukocytes and plasma constituents. Finally, the capillary endothelial cells are highly adapted to underlying tissues, have well developed tight junctions that are tighter than arteries and collecting veins, have more caveolae than arteries and veins, except those found within the blood-brain barrier, and have many phenotypic differences between different vascular beds. DNA microarray studies of endothelial cells cultured from different sites of the human vasculature have revealed differences in the transcriptional profiles between arterial and venous endothelial cells as well as between macrovascular and microvascular endothelial cells. Moreover, differences in growth factor receptor expression have been reported between arterial and venous endothelial cells [27,32,33,34].

In addition to the previously mentioned differences, brain endothelial cells possess unique morphological and functional characteristics [32,33]. The cerebral endothelium is continuous and is characterized by both a lack of fenestration and by the presence of complex tight junctions between endothelial cells which confer much greater resistance than peripheral endothelial cells. Furthermore, brain endothelial cells possess an increased number of mitochondria and a low number of pinocytotic vesicles that limit endocytosis and trancytosis. Endothelial cells in the brain are exposed to numerous astroglial-derived paracrine factors that are essential for the maintenance of the blood brain barrier. These anatomical features allow brain endothelial cells to deliver oxygen, glucose and other nutrients rapidly in order to meet the high metabolic demands of the brain and to participate to the formation of the BBB [35].

Different cellular models from rat, human, mouse, bovine and porcine brain endothelial cells have been used in reports of OGD experiments with the same objective of mimicking as closely as possible the pathological conditions of the vascular system in the ischaemic brain. Most of the studies in which experiments are performed with human brain endothelial cells have used primary culture of human cerebromicrovascular endothelial cells (HCEC) [36,37,38,39,40,41,42,43,44,45] or immortalized human brain capillary endothelial cells (hCMEC/D3) [46,47,48,49]. A recent study by Vukic *et al.* also used primary HBEC cultures and the hCMEC/D3 cell line, but in this case they did not perform OGD experiments. These culture models are used to analyze the molecular signalling pathway responsible of neuroinflamation in Alzheimer’s disease [50]. HCEC are usually characterized by their cobblestone appearance of confluent monolayer, typical of microvascular endothelium in culture, the incorporation of fluorescently labelled acetylated-low density lipoprotein DiI Ac-LDL and high levels of expression of the cerebral endothelium specific enzymes, γ-glutamyl-transpeptidase (γ-GTP) and alkaline phosphatase (ALP). The purity of HCEC cultures is assessed by immunocytochemical staining for factor VIII-related antigen and lack of staining for smooth muscle α-actin. The hCMEC/D3 cell line shows a similar morphology to primary cultures of brain endothelial cells, with confluent monolayers of tightly packed elongated cells that exhibit contact inhibition. This cell line maintains a non-transformed phenotype and the capacity to form a branched network of capillary-like cords on reconstituted extracellular matrix. It constitutively expresses many endothelial and/or BBB markers including the junctional proteins PECAM-1, VE-cadherin, ß- and γ-catenins, zona occludens 1 (ZO-1), junctional adhesion molecule-A (JAM-A), and claudin-5. It has been reported that the hCMEC/D3 cell line recapitulates most of the unique properties of brain endothelium and may thus constitute a well established *in-vitro* model of the human BBB [51]. In the case of Poller *et al.*, this line was used to undertake drug transport studies in this model [52]. In 1997, Muruganandam *et al.* developed an immortalized human cerebral microvascular endothelial cell line (SV-HCEC) as an *in-vitro* model of the BBB [53] although to date they have used this principally to study the properties of the human BBB rather than to conduct OGD experiments.

Unfortunately, human samples, which are best able to mimic *in-vivo* conditions of patients, are often difficult to obtain. For this reason, some authors use other cellular models such as primary rat brain microvascular endothelial cells (rBMEC) from adult and young rats [13,54,55,56,57,58]. The primary rat endothelial cells cultures are identified with factor VIII related antigen, occludins and von Willebrand factor. There also exist different well characterized immortalized rat brain endothelial cell lines, including TR-BBBs, rBCEC4 (conditionally immortalized) and RBE4, GP8/3.9, GPNT, RBEC1. Different studies have used these immortalized cell lines as an *in-vitro* model to perform OGD experiments [48,59,60,61,62,63,64]. These models have made a significant contribution to our current knowledge of the BBB transport function despite the important limitation of having high paracellular permeability to small molecules unlike the BBB. 

Other studies report the use of primary mouse brain microvascular endothelial cells (mBMEC) [14,65,66,67,68,69] as well as mouse immortalized brain endothelial cell lines such as bEnd.3 [12,14,68,70,71,72,73,74,75,76] and MBEC4 [77,78,79]. Other immortalized mouse brain endothelial cell lines include TM-BBB1-5, S5C1-11, bEnd5 although most have been used to perform BBB transport and toxicokinetic studies. mBMEC purity is usually examined based on the expression of factor VIII, vimentin and exhibiting bradykinin receptor function. bEnd.3 line, on the other hand, is obtained from polyoma middle T antigen transformed 6-week mice (BALB/c) brain cerebral cortex. The endothelial nature of the cells is confirmed by the expression of von Willebrand factor and the uptake of fluorescently labelled LDL. Intracellular adhesion molecule 1 (ICAM-1) and vascular cell adhesion molecule 1 (VCAM-1) are constitutively expressed on the cells. The expression of Peyer’s Patch high endothelial receptor for lymphocytes, the mucosal vascular addressin (MAdCAM-1) and E-selectin can be induced on bEnd.3 cells by cytokines and lipopolysaccharide (LPS). 

Some studies have used primary bovine brain capillary endothelial cell (BCEC) culture to perform OGD experiments [80,81,82,83,84,85,86]. Immortalized bovine brain endothelial cell lines such as SV-BEC and t-BBEC117 have also been used in some studies, but in this case, in order to characterize and analyze their BBB properties. BCEC purity is usually evaluated based on the positive expression of factor VIII and vimentin and exhibits the characteristic bradykinin receptors. Primary porcine brain endothelial cells (PBEC) have also been used in different studies that aim to analyse the effect of ischaemia and hypoxia on these brain endothelia cells [48,87,88,89,90].

Finally, isolated brain microvessels, usually from rat [91,92,93,94,95,96,97], mouse [98,99] and gerbil [100,101] submitted to experimental cerebral ischaemia, have been used by different authors. In Table 3A and Table 3B, the most important studies performed with primary and immortalized brain endothelial cells submitted to experimental ischaemia are summarized.

Using primary or immortalized endothelial cell lines has both advantages and limitations. The primary brain capillary endothelial cells have the closest similarity to the BBB *in-vivo* phenotype and exhibit excellent characteristics of the BBB at early passages. However, they are extremely time consuming and costly to generate, are easily contaminated by other neurovascular unit cells (losing their BBB characteristics over passages), rapidly undergo senescence after a limited number of divisions and require a high level of technical skill to perform their extraction from brain tissue. On the other hand, immortalized lines allow the BBB characteristics to be maintained over many passages, permit the formation of functional barriers, are amenable to numerous molecular interventions, are easy to grow, maintain the functionality of some cell transporters, facilitate the transfer of the model between different laboratories, and have a low cost [102]. However, the introduction of immortalizing genes (such as SV40 large T-antigen) may affect a wide variety of cellular functions as they may interact with numerous proteins, altering the physiological cell cycle [103].

### 2.2. Co-Culture

In recent years, the study of the central nervous system has grown to encompass studies of more than a single cell type, recognizing that positive or negative signal transmission exists in different types of brain cells. Signal transmission and coupling among these cells constitute the basis of normal brain function whereas disorderly signal transmission and coupling provides the pathological basis of brain dysfunction and disease [104]. Therefore, in order to better simulate the *in-vivo* pathological conditions of stroke, OGD exposure can be performed on co-cultures consisting of BMECs with other brain glial or neuronal cells. These models represent an excellent tool with which to study the consequences of ischaemic stroke on brain capillaries, neuronal survival and glial function.

#### 2.2.1. Brain endothelial cells and astrocytes

The BBB is a dynamic interface between the blood and the central nervous system that controls the rate of influx and efflux of biological substances needed for the brain metabolic processes and neuronal function. BBB dysfunction and permeability changes (tight junction alterations) are observed in most neurological diseases including stroke [105]. The use of cultured vascular endothelial cells has significant drawbacks. Apart from being time consuming, there is a loss of endothelial cell specific markers following prolonged *in-vitro* culturing, reproducibility of the high electrical resistance found in the BBB in situ is poor, non-endothelial cell contamination is difficult to avoid, and the culture is relatively expensive [106,107]. These disadvantages have led to the development of more reliable *in-vitro* models. Although some investigators have attempted to imitate the *in-vivo* brain environment using glia/endothelial co-cultures (conventional co-cultures or static BBB models) [108,109], others have alternatively attempted to mimic the physiological environment of microvascular endothelial cells by exposing the cells to flow in order to develop a dynamic *in-vitro* BBB model (DIV-BBB). The most commonly used static BBB model consists of a porous membrane support submerged in feeding medium called a Transwell apparatus. The bi-dimensional Transwell system is characterized by a side-by-side diffusion system. Several authors have used this system with different cellular models. Lauer *et al.*, for example, used the permanent microvascular endothelial cell line PBMEC/C1-2 from porcine brains co-cultured with rat C6 glioma cells to perform permeability studies [110]. Malina *et al.* used primary cultures of porcine brain endothelial cells (PBEC) co-cultured in contact and non-contact with rat brain glial cells [111]. Garberg *et al.* compared two primary cell systems: BBEC/astrocytes (bovine brain endothelial cells co-cultured with rat astrocytes) and HPBEC/astrocytes (human primary brain endothelial cells co-cultured with human primary astrocytes) with two transformed cell lines: SV-ARBEC/astrocytes (rat endothelial cells co-cultured with rat astrocytes) and MBEC4 (immoratlized mouse brain microvascular endothelial cells) [112]. Li *et al.* compared four *in-vitro* BBB models: bEnd3 monoculture, bEnd3 co-cultured with astrocytes, co-cultured with two basal membrane substitutes: collagen type I and IV mixture, and Matrigel in its permeability studies [102]. These studies, which were performed to study the permeability properties of the different *in-vitro* models in control conditions, assess BBB function with permeability assays that can be perfomed with fluorescence, isotope labelled tracers such as mannitol glucose and inulin, with TER (transendothelial electrical resistance) measurement, and electric cell-substrate impedance sensing (ECIS) [102].

Other authors have adapted the *in-vitro* BBB cell model to ischaemic conditions by submitting the culture to OGD. Brillault *et al.*, for example, used an *in-vitro* model of the BBB generated by growing bovine brain capillary endothelial cells (BBEC) above rat glial cells to perform permeability studies [82]. When endothelial cells (ECs) were incubated with glial cells, it was found that a shorter OGD time was required to increase EC permeability than for ECs alone. Conditioned medium experiments were also performed in this study, showing that glial cells secreted soluble permeability factors during OGD and so, in response, the ECs become more sensitive to the process. Braillault *et al.* highlighted the importance of the duration of the co-culture (they used 12 days) and the species of glial cells used in the model of co-culture (3-week-old culture containing 60% GFAP-positive cells and 40% non-astrocyte cells) in order to allow a complete differentiation and stabilization of the glial cells. After performing the co-culturing experiments, they separately submitted EC and astrocytes to OGD conditions. Finally, they performed permeability studies on EC incubated with glial conditioned medium. Benchenanek *et al.* also used an *in-vitro* BBB model based on BBMEC and rat glial cells to study tPA transport across the BBB in OGD conditions [81]. Similarly, some authors have used BBMEC cultured with astrocyte conditioned medium (from C6 cells) as an *in-vitro* BBB model [80,85]. Other recent studies by Allen *et al.* have aimed to explore in a co-culture consisting of human brain endothelial cells with human astrocytes whether small GTPase RhoA and its efector protein Rho kinase are involved in permeability changes mediated by OGD [36]. Mysiorek *et al.* used rat brain capillary endothelial cells with rat glial cells to study whether peroxisome proliferator-activated receptor α (PPAR-α) activation acts on BBB permeability in ischaemic conditions [113]. Finally, Cowan and Easton used the immortalized human brain endothleial cell line hCMEC/D3 to study the effect of neutrophils on the increase in BBB permeability associated with ischaemia. This study proved that OGD induces reversible increases in the cell line permeability and showed that neutrophils mitigate increases in permeability [46].

On the other hand, the DIV-BBB originated from a modification of a cell culture system that was used for hybridoma cell expansion. This three-dimensional system provides quasi-physiological experimental conditions for culturing endothelial cells and astrocytes in a capillary-like structure consisting of hollow fibre tubes (mimicking capillary vessels) inside a sealed chamber (representing the extraluminal space) that enables co-culturing of endothelial cells with extraluminally seeded glia, where endothelial cells develop a morphology that closely resembles the endothelial BBB-specific phenotype (low permeability, negligible extravasation of proteins, expression of glucose transporter, expression of BBB-specific ion channels) in situ as they are exposed to shear stress [107,114]. The entire apparatus resides in a water-jacketed incubator with 5% CO2 and can be sterilely sampled by moving it inside a laminar flow hood. Different studies have used bovine aortic endothelial cells (BAEC) co-cultured with C6 rat glioma cells in a dynamic BBB model [107,114,115,116,117,118]. Cucullo *et al.* compared four different human endothelial cell types: AVM-ECs (arteriovenous malformations surgically removed from patients), HUVEC and HBMEC (primary) and HCMEC/D3 (immortalized), co-cultured with human astrocytes. They concluded that the HCMEC/D3 cell line maintains the *in-vitro* physiological permeability barrier properties of the BBB, even in the absence of abluminal astrocytes [47]. In a recent study, Cucullo *et al.* have presented an improved DIV-BBB model composed of normal adult human brain microvascular endothelial cells and human adult astrocytes to study how haemodynamic changes and systemic inflammation affect the integrity of the brain microvasculature. This new model has the novelty of having transcapillary microholes to enable transendothelial cell trafficking between the vascular and the parenchymal compartment [119]. 

The advantages and limitations of each type of BBB model together with some of the most significant authors that have used the corresponding models are set out in Table 4.

#### 2.2.2. Brain endothelial cells and neurons

Given the lack of a close anatomical connection between BMECs and neurons, it used to be assumed that their relationship was indirectly mediated by astrocytes [57]. However, a study published in 2003 by Carmeliet *et al.* [120] provided strong evidence for the direct functional correlation between BMECs and neurons. On investigating the hemodynamic neurovascular coupling mechanisms and the relationship between neurogenesis and angiogenesis, it was found that there was communication between endothelial cells and neurons by mutual coordination both in the regulation of local cerebral blood flow and the initiation of repair following injury. Many more recent studies have also demonstrated the relationship between BMECs and neurons, but most have only examined these connections in physiological conditions. Li *et al.* aimed to elucidate the effect of paracrine signalling of rat BMECs in pathological conditions on rat cortical neuron survival. Normal and injured neurons were treated with EC-N-CM (endothelial cells normal conditioned medium) and EC-I-CM (endothelial cells ischaemia conditioned medium), respectively, in order to find the relationship between the two kinds of cells in both the physiological state and cerebral ischaemia. The authors found that EC-N-CM had no apparent role on the normal neurons, but had a neuroprotective effect on injured neurons. Once endothelial cells were injured, the neuroprotective effect disappeared and in fact became neurotoxic. When the ischaemic injury was initiated, the neurotoxic effect was found to further induce the injury on neurons and to promote neuron death. Paracrine signalling of BMECs was concluded to play a role in the survival of neurons, and that this role changed depending on whether the conditions were normal or pathological [57].

Roitbak *et al.* used a direct and a Transwell co-culture model with mouse neural stem/progenitor cells (NSPC) and mouse brain endothelial cells (bEnd.3) to examine the interactions between the two cellular types in the context of ischaemia both *in-vitro* and *in-vivo* (NSPC intracerebral mice transplantation subjected to transient middle cerebral artery occlusion). To this end, cells were submitted to three hours of OGD using deoxygenated glucose-free Earl’s Balanced Salt Solution [74]. This study lends support to NSPCs having a proangiogenic role under ischaemic conditions through HIF-VEG signalling pathways.

### 2.3. In-Vitro Ischaemic Media

Different oxygen-glucose deprivation media are currently being used to perform *in-vitro* ischaemic experiments with microvascular endothelial cells (see Table 5). Glucose-free Krebs solution is a common buffer used in OGD experiments to mimic *in-vitro* brain ischaemic conditions. It was first used by Pedata *et al.* in ischaemic experiments with hippocampal slices [121] and has since been used to submit rat [55,57] and human [42,43,45] BMECs to ischaemic conditions.

Balanced salt solutions, based on the physiological saline solution first developed by Ringer, are also used as ischaemic medium to submit cerebrovascular endothelial cells to OGD experiments. These solutions are composed of inorganic salts which maintain cells in a viable state rather than promoting growth. Glucose-free Hanks’ Balanced Salt Solution (HBSS) is a commercially available medium buffered with phosphate that contains the salt composition indicated in Table 5, although this can vary slightly from one manufacturer to another. This solution contains the same inorganic salts as glucose-free Krebs solution, although the concentrations vary, and additionally contains magnesium sulphate (MgSO_4_·7H2O) and anhydrous dibasic sodium phosphate (Na_2_HPO_4_). 

Several authors have used deoxygenated glucose-free HBSS to mimic *in-vitro* ischaemia-like conditions in mouse cerebral vascular endothelial cells [65,67,69,99], bovine cerebral endothelial cells [86] and human brain microvascular endothelial cells (HBMEC) [36,122].

Another balanced salt solution also used in the cerebromicrovascular endothelial cells OGD model is deoxygenated glucose-free Earl’s Balanced Salt Solution (EBSS). The basic formulation consists of potassium chloride (KCl), sodium bicarbonate (NaHCO_3_), sodium chloride (NaCl), and sodium phosphate monobasic (NaH_2_PO_4_·H_2_O) but additional components may be found. Domoki *et al.* and Roitbak *et al.* performed the OGD experiments with the EBBS medium using rat and piglet cerebromicrovascular endothelial cells and mouse brain endothelial cells, respectively, as ischaemic *in-vitro* models [54,74].

Recent studies such as Jung *et al.* and Guo *et al.* also used a deoxygenated glucose-free balanced salt solution but with slightly different concentrations and compositions of the inorganic salts (without MgCl_2_ and KH_2_PO_4_ but with NaH_2_PO_4_ and HEPES) [12,70]. This medium, called BSSO.O as it consists of 0.0 mM of glucose. The salt composition can be annoted in table 5. Immortalized mouse brain endothelial cell lines (bEnd.3) were used in the two studies. 

A deoxygenated glucose-free DMEM solution has been used in various studies with different animal models, including primary rBMECs [13], mouse brain endothelial cell culture (mBMEC and bEnd.3) [14,71,73,123] and a human brain endothelial cell line (hCMEC/D3) [46].

Brillault *et al.* and Culot *et al.* performed OGD experiments with bovine brain capillary endothelial cells (BBECs) and used a serum-lightened (5% v/v serum) and low-glucose (0.2 g/L) medium equilibrated with nitrogen in order to mimic the ischaemic conditions. They used this serum-lightened medium to avoid temporary permeability-increasing artefacts due to serum growth factors as their aim was to investigate the effect of ischaemia on permeability changes [82,83].

Finally, Dehouck *et al.*, Abbruscato *et al.*, Slevin *et al.*, and Paulson *et al.* submitted human (HBMEC) [41] and bovine (BCEC) [80,84,85] brain microvessel endothelial cells to OGD using conventional medium without glucose, previously equilibrated in the hypoxic chamber. Similarly, Milner *et al.* and Benchenane *et al.* exposed primary cultures of murine [66] and bovine [81] brain endothelial cells to OGD using conventional medium with low glucose concentrations and also equilibrated in a hypoxic chamber.

### 2.4. Time of OGD Treatment

In order to mimic the ischaemic conditions as closely as possible, it is important to fix and determine the optimal period of time that cerebromicrovascular endothelial cells will be submitted to OGD conditions. This will vary depending on the cellular type, the ischaemic medium used and, especially, the purpose of the study.

As previously reported, PC (with brief, non-lethal ischaemia) can reduce the injury of subsequent severe ischaemia. This phenomenon described in several organs is called ischaemic tolerance. Ischaemic PC stimuli can be either a short period of global ischaemia or a short period or periods of focal cerebral ischaemia. Some authors use the PC protocol in combination with OGD, in order to elucidate the role of this process plays in the decrease of cell injury after OGD treatment. *In-vitro* ischaemic PC studies have allowed the use of such cultures to examine the mechanisms involved in this process. 

As the duration of OGD treatment varies widely between different studies, principally depending on the aim of the study, we decided to review this issue categorizing the reported studies by species and cell type. However, although for ease of reference we have made this classification, it should not be understood that the cellular model used in any way determines the length of the OGD treatment.

#### 2.4.1. Rat endothelial cells

Li *et al.*, who used Krebs buffer to mimic the OGD model with rBMECs, studied the effect on neuron survival of paracrine signalling under pathological conditions, testing different periods of OGD (1 h,2 h, 4 h, 6 h and 8 h) and checking endothelial cell viability by the MTT [(3-(4,5-dmethylthiazol-2-yl)-2,5-diphenyltetrazolium bromide)] assay. They finally selected to use 6 h of OGD as this was the first condition in which a significant decrease (P < 0.001) of cell viability was observed in comparison with the control group (not exposed to OGD) [57]. Similarly, Du *et al.* used the same ischaemic medium and cells but different periods of OGD (1, 3, and 16 h) to study the involvement of placental growth factor (PIGF) in cerebral ischaemia injury [55].

An also used a rBMEC model but with DMEM glucose-free solution. The cells were submitted to a previous PC protocol consisting of a 30 min exposure to OGD and normoxic incubation for 24 h for reoxygenation before inducing OGD injury for 2.5 h. A second group was studied without the PC protocol to compare the three conditions (control, OGD, PC + OGD). The PC procedure was included in order to analyze the effects of PC on the tight junction and cell adhesion of cerebral endothelial cells. Their results demonstrated that OGD preconditioning alleviated the injury and induced protective effects on endothelial cells. Transendothelial electrical resistance was similar to the control, ZO-1 and F-actin localization were maintained as in the control, and a reduction of ICAM-1 and VCAM-1 expression (involved in leukocyte adhesion and activation) was observed. In their preliminary experiments, the authors found on testing different periods of exposure to OGD (15 min, 0.5 h, 1 h, 1.5 h, 2 h, 2.5 h, 3 h) that more than 2 h produced irreversible injury, 3 h produced severe damage with apoptosis or necrosis in most cells, whereas 0.5 h of PC did not result in apparent injury but led to ischaemic tolerance [13]. 

Domoki *et al.*, who used EBSS medium and rat cerebromicrovascular endothelial cells (CMVECs), performed ischaemic experiments exposing the cells to 12 h of OGD. The aim of the study was to determine whether primary cultures of rat CMVECs were sensitive to L-glutamate (L-glut) or *N*-methyl-D-aspartame (NMDA). To this end, they combined L-glut/NMDA (0.1-1 mM) treatment with OGD for 12 h. After OGD, EBSS was replaced with regular culture medium for 12 h before determining cellular viability. The exposure of rat CMVEC cultures to 12 h of OGD was found to significantly reduce viability. However, the addition of 1 mM L-glut or NMDA into the glucose-free EBSS solution did not affect cell death induced by OGD [54]. On the other hand, there are also some authors that used primary [56,58] or immortalized human brain endothelial cells in their studies, but in these cases, they performed hypoxia (oxygen deprivation) instead of ischaemia experiments (oxygen and glucose deprivation) (see Table 3A and Table 3B). In summary, when rat brain endothelial cells are used as the OGD model, the time of OGD exposure varies greatly (from 1 to 16 hours) depending on the medium used and the objective of the study. 

#### 2.4.2. Mouse endothelial cells

When the cerebrovascular endothelial cells are from mouse, OGD exposure times seem to be more consistent between the different studies. Yin *et al.*, for example, investigated the potential interaction of ataxia telangiectasia mutated (ATM) and NF-KB after OGD in mouse cerebral endothelial cells, exposing cells with deoxygenated glucose-free HBSS to OGD for 4 h. They performed the analysis at 4 h after OGD onset, as this was approximately when 30% of cells had died and when ATM was induced [69]. The same authors exposed mouse endothelial cells to 4 and 16 h of OGD (with deoxygenated glucose-free HBSS) in a more recent study [99] to study the molecular mechanisms of peroxisome proliferator-activated receptor δ (PPARδ) on ischaemia-induced cerebrovascular injury. Taking 4 and 16 h of OGD exposure, the authors explored whether Golgi fragmentation, an essential process in the development of cell death [124] occured in OGD. They report that after OGD the morphology of the Golgi apparatus changes to debris-like structures scattered in the cytoplasm (nuclear shrinkage). These alterations in cellular morphology were observed as early as 4 h after OGD onset and were further enhanced after 16 h.

Hu *et al.* and Narasimhan *et al.* submitted primary mouse cerebral endothelial cells to 4 h and 8 h of OGD, respectively, with glucose-free buffered salt solution (HBSS) [65,67]. Milner *et al.* submitted the cerebral cells to 18 h of OGD using low-glucose medium (1.0 g/L glucose) [66,125]. Other recent studies that used mouse immortalized cerebral endothelial cells (b.End3) to perform the OGD experiments exposed the cells to glucose-free DMEM medium during 3, 6, 12, 18 or 24 h [123] and 5 h [73] of OGD or to deoxygenated glucose-free BSS0.0 during 60 min, 90 min, 3 h or 6 h of OGD, depending on the experiment [12], or 6 h of OGD [70] to mimic ischaemic conditions. Yan *et al.*, also used the b.End3 cell line, but in this case they used serum-free low-glucose medium during larger periods of time (24 h, 36 h, 48 h) as the OGD protocol [76].

Finally, Andjelkovic *et al.* exposed an immortalized mouse brain endothelial cell line (bEnd.3) and primary cultures of mBMEC to 5 hours of OGD using deoxygenated DMEM glucose-free medium to examine whether cerebral endothelial cells can be preconditioned *in vitro* in the absence of other cell types. This study consisted of four different sets of experiments comparing different PC duration, the effects of altering the time interval between the PC stimuli and OGD injury, the cell viability of the two types of cerebral endothelial cells and the effect of PC on the endothelial intercellular adhesion molecule (ICAM-1) mRNA and protein after OGD. In all of the experiments cerebral endothelial cells were exposed to a short duration of OGD as a stimulus and, after different time intervals, exposed to a longer duration of OGD (5 h) with or without reoxygenation to examine whether prior PC would reduce cell injury. In the first set of experiments they examined the optimal duration of the PC (15, 30, 45, or 60 min) stimulus using bEnd.3 using lactate dehydrogenase (LDH) assays. In this study the authors found evidence of protection from 15 min of OGD as the PC stimulus to reach the maximum protection level between 45 and 60 min of OGD (longer periods were not used as they were found to cause endothelial damage). In the second set of experiments, the duration of the PC stimulus was kept constant (1 h), and the time interval between that stimulus and the prolonged OGD was varied (4 h, 12 h, 24 h, 48 h, 72 h). They found that 1 h of PC stimulus and a 24 h interval between the PC stimulus and the OGD injury were the optimal conditions to provide a protective effect on bEnd.3. This delay was considered to be due to the need for new protein synthesis [126], and this also appears to be the case for the protection of the cerebral endothelium. The third set of experiments compared the effects of PC on cell viability (using calcein-AM and EthD-1) of two types of cerebral endothelial cells, bEnd.3 and primary cultures (mBMEC). The two types of cerebral endothelial cells gave almost identical results when they were exposed to 1 h of PC stimuli and 24 h as the time interval between PC and the 5 h of OGD. The PC was found to significantly reduce LDH release in both cell types. To determine the effect of PC on the inflammatory response of bEnd.3 cells, the fourth set of experiments examined the mRNA and protein levels of the ICAM-1 after PC stimulus (15, 30, 45, 60 min) and OGD with reoxygenation (24 h, 48 h, 72 h). They conclude that PC has anti-inflammatory effects on cerebral endothelial cells, as they observed a down-regulation of ICAM-1 increased when the duration of the PC was lengthened from 15 to 60 min [14].

In conclusion, the time of OGD used in the experiments with mouse cerebromicrovascular endothelial cells normally ranges from 4 to 8 h, with the exception of the previously reported studies performed by Lee *et al.* and Yan *et al.* which used significantly longer periods of time (24 h, 36 h, 48 h).

#### 2.4.3. Human cerebral vascular endothelial cells

Human cerebromicrovascular endothelial cells (HCEC) have also been used as an *in-vitro* model to mimic ischaemic conditions. Zhang *et al.* and Stanimirovic *et al.*, who performed experiments exposing cells to glucose-free Krebs solution during 4 h, are a clear example [42,43]. Authors such as Zhang *et al.* exposed HBMEC to longer periods of time (8 hours) using HBSS without serum and performing a previous hypoxic preconditioning protocol consisting of two 3 h periods of hypoxia/day (with an interval of 1 h of normoxia) during five days [122]. Other recent studies by Slevin *et al.*, Guo *et al.*, Allen *et al.* and Cowan and Easton, have also used human brain endothelial cells in their experiments [36,38,41,46]. In the cases of Slevin *et al.* and Guo *et al.*, 6 and 24 h of OGD were used, respectively, and in the other cases different periods of *in-vitro* ischaemia were selected. Allen *et al.* used either 4 h or 20 h (using HBSS medium), and in the case of Cowan and Easton cells (in which they used hCMEC/D3, an immortalized human brain endothelial cell line) were exposed for 1 h, 12 h and 24 h to OGD in order to compare results in the different conditions. In the case of Cowan and Easton, two different OGD media, glucose-free PBS and glucose-free DMEM, were used.

Like the experiments performed with rat brain endothelial cells, the OGD experiments with human cerebral endothelial cells exposed the cells to a wide variety of different periods of time, ranging from 4 or 8 h to 24 h.

#### 2.4.4. Bovine endothelial cells

Some authors have also used bovine brain capillary endothelial cells for their experiments. Brillault *et al.*, for example, aimed to investigate the effect of hypoxia on permeability changes by specifically exposing cells to serum-lightened (5% v/v serum) and low-glucose (0.2 g/L) medium during 4 h to mimic *in-vitro* ischaemic conditions [82]. Similarly, Culot *et al.* used the same medium as well as the same time of OGD as Brillault *et al.* [83]. On the other hand, Abbruscato *et al.* and Paulson *et al.* used conventional medium without glucose during 6 hours as the OGD protocol in their experiments with bovine brain capillary endothelial cells [80,85]. The purpose of both studies was to explore different molecular mechanisms of nicotine and tobacco smoke effects through the BBB during ischaemia. On the other hand, Benchenane *et al.* used a serum-free and low-glucose medium during 4 h to determine whether vascular tPA crosses the blood-brain barrier (BBB) during cerebral ischaemia [81]. Finally, Xu *et al.* perform the OGD experiments using HBSS medium during different periods of time between 1-8 hours. The aim of the study was to characterize bovine cerebral endothelial cell death in relation to iNOS expression after experimental ischaemia [86]. In summary, the different studies with bovine brain endothelial cells used a period of OGD ranging from 1-9 h, although most were within the 4-6 h range.

#### 2.4.5. Porcine endothelial cells

Several studies have used porcine brain endothelial cells as a BBB cellular model although most perform hypoxia rather than undertaking ischaemic experiments. Beetsch *et al.* used several periods of hypoxia, none less than 10 h, to measure oxygen free radical production by cerebral endothelial cells and determine its role in hypoxia injury [87]. Fischer *et al.* used periods from 1.5 to 48 h of hypoxia to investigate the effects of methohexital and thiopental on the permeability of the endothelial cell monolayer under these conditions [89]. In another study, the same group used 24 h of hypoxia using the same *in-vitro* BBB model to evaluate the mechanisms by which hypoxia regulates paracellular permeability. They concluded that the VEGF released leads to delocalization, decreased expression and enhanced phosporylation of ZO-1 (zona occludens) [48]. The time of hypoxia treatment in the studies cited here vary widely between the different reports from 1.5 to 48 h.

### 2.5. Time of Reperfusion

Although the previously reported studies are based on the OGD experimental model which consisted of replacing the conventional medium and exposing the cerebral endothelial cells from different animal models to OGD conditions, they principally differ in the reperfusion time during which sample collection was performed. All the authors analyzed the samples from between 16 and 20 h of reperfusion, except one who used 72 h of reoxygenation [14], when a single collection was made [36,38,70,86,127] and from 0 to 24 h when a time course analysis was performed [43,69,73,99].

## 3. Discussion and Conclusions

Ischaemic cerebral stroke is produced by arterial occlusion resulting in dramatically reduced levels of oxygen and glucose to the region supplied. In recent years, cerebral endothelium, an important component of the neurovascular unit, has become a potential therapeutic target for neurological diseases including stroke [12,105,128,129,130].

Different studies aiming to mimic the ischaemic conditions of stroke have used hypoxia as well as ischaemic models. For *in-vitro* experiments, OGD is the closest approximation to the conditions that occur during cerebral ischaemia as only oxygen deprivation is performed in hypoxia experiments. For this reason we have focused on ischaemic models in reviewing the different published studies that have used cerebral vascular endothelial cells as an *in-vitro* ischaemic cellular model. 

The studies that have been reviewed here reveal that there is no general consensus as to the conditions (ischaemia medium, time of OGD treatment and time of reperfusion) and cellular models to be used in the OGD experiments performed with cerebrovascular endothelial cells. However, the most widely used cellular model in the last thirteen years has been that of the mouse cerebral endothelial cells, principally the bEnd.3. In this case, we have seen that there is a greater correlation in the type of ischaemia medium used than in the OGD exposure time. The most common media used in OGD experiments with these cells are especially glucose-free HBSS but also DMEM glucose-free. The time of OGD varies widely between studies although it is more standardized when glucose-free HBSS is used. In contrast with the studies reported using mouse endothelial cells, in which OGD conditions appear to be more homogeneous, the experiments performed with rat as well as human brain endothelial cells use very different ischaemia media (including glucose-free Krebs solution, glucose-free HBSS, glucose-free EBSS, glucose-free DMEM, glucose-free conventional medium) and a wide range of times of OGD exposure. Therefore, although it appears that for some species there is a consensus in some reviewed studies, when OGD experimental conditions and protocol design are being developed (ischaemia medium and especially the time of OGD treatment) it is extremely important to take the objectives of the study into account.

Most authors performed their experiments with brain microvascular endothelial cells to study ischaemia given that they are responsible for angiogenesis in cerebral ischaemia. However, some authors used HUVEC (human umbilical vein endothelial cells) cultures in their experiments as they are a cellular model in angiogenic studies [131]. These cells are a rich source of CD34+ stem cells, which have been found to secrete numerous angiogenic factors, including VEGF, HGF and IGF. Despite this, it has been reported that ECs from different blood vessels and microvascular ECs from different tissues have distinct and characteristic gene expression profiles [132,133] and so this model cannot supply reliable information when the focus is brain ischaemia research since the differential gene expression profiles of ECs from different sites affect their development potential, their interaction with other cells and their response to experimental manipulation. It is therefore necessary to use organ specific endothelial cells in the *in-vitro* studies. 

Although most of the studies reviewed used a serum-free media in models of ischaemia, some used a serum-lightened medium in their experimental studies in order to avoid temporary permeability-increasing artefacts due to serum growth factors and improve cell viability and proliferation amongst other reasons. These differences in the media composition should be taken into account in the analysis of the results and in comparing different studies, as serum growth factors has been reported as affecting the barrier properties of an endothelial cell culture, promoting a decrease in the electrical resistance and the opening of the tight junctions, thus affecting the final differentiation of the cells [134,135].

Some of the studies reported performed the OGD experiments co-culturing endothelial cells with neurons or astrocytes in order to mimic as closely as possible *in-vivo* brain conditions (BBB). More reliable results are obtained in this way as they take into account paracrine signalling among the different cell types as occurs in physiological and physiopathologic conditions. Hence, *in-vivo* ischaemic conditions are better simulated and the repercussions of injury to the different brain cell types can be better analyzed. With regards to the brain endothelial cell differential response in mono versus co-culture conditions, it has been reported that when brain endothelial cells are assayed in co-culture with astrocytes, an earlier increase in the permeability of the ECs (hyperpermeability) is observed in OGD experiments in comparison with EC monocultures. This occurs due to glial cell activation that produces a secretion of soluble factors which make endothelial cells more sensitive to OGD. Some of the reported studies used conditioned media from endothelial cells previously exposed to OGD conditions or from control conditions with cultured neuronal cells to study the effect of this interaction on neuronal [57] and non-neuronal [59,82] brain cells surveillance (astrocytes, oligodendrocyte precursor cells). In these cases, instead of co-culturing the two cell types, the model tries to mimic the co-existence of different injured and non-injured cell types, which may occur in some brain areas after stroke. A recent study into the new dynamic human BBB model developed by Cucullo *et al.* is particularly worthy of mention. This new DIVBBB system is composed of human endothelial cells and human astrocytes, which avoid species differences in the co-culture providing a more reliable physiological model. As this *in-vitro* model allows cell extravasation analysis under realistic perfusion pressures and shear stress levels, it is a useful tool to study the BBB characteristics in different pathophysiological conditions.

It should be noted that the literature reviewed here does not provide viability, mortality, gene expression data of cerebral endothelial cells exposed to OGD after longer than 72 h post *in-vitro* ischaemia. In our opinion, further studies are necessary to better understand the long-term response of microvascular endothelial cells after OGD as part of a broader attempt to unravel the different molecular mechanisms of stroke. Finally, we would like to stress the importance of standardizing the OGD experimental conditions with cerebrovascular endothelial cells in order to provide comparative and reproductive results and to avoid misinterpretation due to the cellular model and experimental conditions used.

## Figures and Tables

**Table 1:** The events occurring in different phases of angiogenesis and the participating molecules.

Phase	Events		Participating molecules
1	Existing vessels dilate, vascular permeability increases and exracellular matrix is degraded.		NO, VEGF, PECAM-1, VE-cadherin, Ang1 and MMPs.
2	Endothelial cells proliferate and migrate as physical barriers are dissolved.		VEGF, angiopoietins, FGFs, integrins, PECAM, Eph/ephrin receptor-ligand pairs, VE-cadherin and members of the connexin family
3	Endothelial cells assemble, form cords and acquire lumens.		VEGF, angiopoietins, integrins.
4	Long-term survival of vascular endothelium: once new vessels have assembled, the endothelial cells become resistant to exogenous factors (quiescent).		p53, p21, p16, p27, Bax and p42/44 mitogen activated protein kinase.
5	Vascular endothelium is differentiated to meet local needs.	BBB	Interaction between GFAP, pericytes and angiotensin.
		Tight junctional complex	Cadherins, occludins, guanylate kinase homologous proteins

Nitric oxide (NO), vascular endothelial growth factor (VEGF), platelet/endothelial cell adhesion molecule-1 (PECAM-1), vascular endothelial cadherin (VE-cadherin), angiopoietin-1 (Ang1), matrix metalloproteinases (MMPs), fibroflast growth factors (FGFs), GFAP (glial fibrillary acidic protein).

**Table 2:** Advantages and limitations of the most important *in-vitro* angiogenesis assays used with endothelial cells and organ culture.

	Type of assay	Specific assay	Advantages	Main limitations
**Endothelial cells**	Matrix degradation	Matrix invasion assay	-High sensitivity -High reproducibility -Allow precise quantifications -Control of the different parameters involved in the angiogenesis -Ability to study individual steps of angiogenesis	-Technical difficulty of setting up the assays -Difficulty of comparing different studies due to the diversity of reagents used
	Proliferation	MTT assay Thimidine incorporation BrdU incorporation Cell cycle analysis		
	Apoptosis	TUNEL assay Annexin V assay		
	Migration	Boyden chamber assay phagokinetic track wound healing under agarose assay		
	Differentiation and morphogenesis	Matrix assay on Matrigel Matrix assay on collagen/Matrigel collagen sandwich 3D gel co-culture		
**Organ culture assay**	Aortic and carotid ring assays	Rat aortic ring Mouse aortic ring Chick aortic arch Porcine carotid artery	-Closest mimicking of the *in-vivo* situation as the surrounding non-endothelial cells and supporting matrix are included -Endothelial cells are quiescent in explant tissue, as *in-vivo* angiogenesis	-Non-human tissues -Large vessels assays are far from being ideal *in-vivo* angiogenic conditions -Explant tissue from growing embryo does not preserve the endothelial cells in a quiescent state
	Tissue from growing embryos	Placental vein disk Fetal bone explants: Mouse metatarsal assay		
	Embryoid body assay			

**Table 3A:** Studies performed with primary brain endothelial cells submitted to experimental ischaemia.

Origin	Specific line	Characteristics and purity assessment	Authors
Human	HCEC	-Cobblestone appearance -DiI Ac-LDL uptake -γ-GTP, ALP high expression -Staining for factor VIII related antigen, no α-actin	Stanimirovic *et al.* 1997 [42] Howard *et al.* 1998 [40] Zhang *et al.* 1999 [43] Zhang *et al.* 2000 [44] Griffin *et al.* 2004 [37] Slevin *et al.* 2009 [41] Allen C *et al.* 2010 [36] Guo S *et al.* 2010 [38] Haarmann *et al.* 2010 [39] Zhu *et al.* 2010 [45]
Rat	rBMEC	-Factor VIII related antigen, occludins and von Willebrand factor expression	Hiu T *et al.* 2008 [56] Domoki *et al.* 2008 [54] Liu *et al.* 2008 [58] An *et al.* 2009 [13] Li *et al.* 2009 [57] Du *et al.* 2010 [55]
Mouse	mBMEC	-Expression of factor VIII, vimentin -Exhibition of bradykinin receptor function	Andjelkovic *et al.* 2003 [14] Yin *et al.* 2002, 2010 [69,99] Hu *et al.* 2006 [65] Yang *et al.* 2007 [68] Milner *et al.* 2008 [66] Narasimhan *et al.* 2009 [67]
Bovine	BCEC	-Positive expression of factor VIII and vimentin -Exhibits the characteristic bradykinin receptors	Xu *et al.* 2000 [86] Brillault *et al.* 2002 [82] Dehouck *et al.* 2002 [84] Abbruscato *et al.* 2004 [80] Benchenane *et al.* 2005 [81] Paulson *et al.* 2006 [85] Culot *et al.* 2009 [83]
Porcine	PBMEC	-Factor VIII related antigen, vimentin, laminin, ACE expression -Negative expression for α-actin	Beetsch *et al.* 1998 [87] Fischer *et al.* 1996 [89] Fischer *et al.* 2002 [48] Fischer *et al.* 2004 [90] Elfeber *et al.* 2004 [88]

**Table 3B:** Studies performed with immortalized brain endothelial cells submitted to experimental ischaemia.

Origin	Specific line	Characteristics and purity assessment	Authors
Human	hCMEC/D3	-Maintains a non-transformed and a contact-inhibition phenotype -Branched network on extracellular matrix -BBB markers: PECAM-1, VE-cadherin, ß- and γ-catenins, ZO-1, JAM-A, claudin-5	Cowan *et al.* 2010 [46] Cucullo *et al.* 2008 [47]
Rat	RBE4	-Contact-inhibited monolayer, spindle-fibre shape -Factor VIII, E-cadherin, ICAM-1, Endothelin-1 expression	Rabbin *et al.* 1996 [62] Chow *et al.* 2001 [60] Fischer *et al.* 2002 [48] Yu *et al.* 2007 [64]
	GPNT	-Spindle-fibre shape -Factor VIII, α,Β and γ-catenin, ICAM-1, VCAM-1expression -AcLDL uptake	Robertson *et al.* 2009 [63]
	rBCEC4	-Contact-inhibited monolayer, endothelial cell shape -Factor VIII, ACE expression	Davis *et al.* 2010 [61]
Mouse	bEnd.3	-Positive expression of von Willebrand factor, ICAM, VCAM -Uptake of fluorescent labelled LDL -Peyer’s Patch high endothelial receptor, MadCAM-1, E-selectin induction by cytokines and LPS	Andjelkovic *et al.* 2003 [14] Li *et al.* 2006 [72] Yang *et al.* 2007 [68] Roitbak *et al.* 2008 [74] Tixier *et al.* 2008 [75] Yan *et al.* 2008 [76] Lee *et al.* 2009 [71] Plane *et al.* 2010 [73] Guo *et al.* 2010 [12] Jung *et al.* 2010 [70]
	MBEC4	-Uptake of fluorescent labelled LDL -ALP and γ−GTP activity	Yamaji *et al.* 2003 [79] Dohgu *et al.* 2007 [77] Nishioku *et al.* 2007 [78]

Angiotensin-converting enzyme (ACE), acetylated low density lipoprotein (AcLDL), intercellular adhesion molecule (ICAM), vascular cell adhesion molecule (VCAM), mucosal addresin cell adhesion molecule (MadCAM-1), alkaline phospatase (ALP), γ-glutamyl transpeptidase activity (γ-GTP), lipopolysaccharide (LPS).

**Table 4:** Advantages and limitations of the different *in-vitro* BBB models and some representative studies.

	Authors	Advantages	Limitations
**BBB static model (Transwell system)**	- Brillault *et al.* 2002 [82] -Abbruscato *et al.* 2004 [80] -Benchenanek *et al.* 2005 [81] -Paulson *et al.* 2006 [85] -Mysiorech *et al.* 2009 [113] -Allen *et al.* 2010 [36] -Cowan *et al.* 2010 [46]	-Low cost -Repeatability -Feasibility of performing high-throughput screening	-Lack of physiologic shear stress due to absence of intraluminal flow (endothelial cell differentiation and loss of BBB phenotype) -The tightness measured by TEER and permeability is much less stringent than *in vivo* -The endothelial cells pile up in a multilayer fashion due to lack of antimitotic influences by laminin and flow
**BBB dynamic model**	-Stannes *et al.* 1996 [116] -Stannes *et al.* 1997 [117] -Pikney *et al.* 1998 [115] -Stannes *et al.* 1999 [118] -Santaguida *et al.* 2006 [107] -Cucullo *et al.* 2002 [114] -Cucullo *et al.* 2010 [119]	-Endothelial cells grow with flow as in *in-vivo* conditions -Enables real-time continuous monitoring of BBB functions (via electrodes inserted in the luminal and abluminal compartments) -Allows the study of the response to different shear stress: brain capillaries to larger vessels -Allows on-line computer BBB integrity assessment (TEER monitoring )	- The system does not allow the study of the effects of turbulent or otherwise altered flow - The model does not include all the elements of the brain environment (neurons, microglia, *etc.*) -The hollow fibre wall (basal membrane) has greater thickness than vessels in the brain

**Table 5:** Composition of the different media used in the different studies performed with brain endothelial cells submitted to OGD.

OGD	Composition/description	Species	Authors
Glucose-free Krebs solution	NaCl (119 mM), KCl (4.7 mM), KH_2_PO_4_ (1.2 mM), NaHCO_3_ (25 mM), CaCl_2_ (2.5 mM), and MgCl_2_ (1 mM)	Human	Stanimirovic *et al.* 1997 [42] Zhu *et al.* 2010 [45]
		Rat	Zhang *et al.* 1999 [43] Li *et al.* 2009 [57] Du *et al.* 2010 [55]
Glucose-free HBSS	CaCl_2_ (1.26 mM), MgCl_2_-6H_2_O (0.493 mM), MgSO_4_-7H_2_O (0.407 mM), KCl (5.33 mM), KH_2_PO_4_ (0.441 mM), NaHCO_3_ (4.17 mM), NaCl (137.93), and Na_2_HPO_4_ (0.338 mM)	Human	Zhang *et al.* 2007 [122] Allen *et al.* 2010 [36]
		Mouse	Yin *et al.* 2002 [69] Hu *et al.* 2006 [65] Narasimhan *et al.* 2009 [67] Yin *et al.* 2010 [99]
		Bovine	Xu *et al.* 2000 [86]
Glucose-free EBSS	KCl, NaHCO_3,_ NaCl, NaH_2_PO_4_-H_2_O additional components may be found	Mouse	Roitbak *et al.* 2008 [74]
		Rat	Domoki *et al.* 2008 [54]
Glucose-free BSSO.O	NaCl (116 mM), CaCl_2_ (1.8 mM), MgSO_4_ (0.8 mM), KCl (5.4 mM), NaH_2_PO_4_ (1 mM), NaHCO_3_ (14.7 mM), and HEPES [4-(2-hydroxyethyl)-1-piperazineethanesulfonic acid] (10 mM, pH 7.4)	Mouse	Jung *et al.* 2010 [70] Guo *et al.* 2010 [12]
Glucose-free DMEM solution	Dulbecco’s modified Eagle’s medium without glucose	Human	Cowan *et al.* 2010 [46]
		Mouse	Andjelkovic *et al.* 2003 [14] Lee *et al.* 2009 [71] Plane *et al.* 2010 [73] Lee *et al.* 2010 [123]
		Rat	An *et al.* 2009 [13]
Serum-lightened medium	serum-lightened (5% v/v serum) and low-glucose (0.2 g/L)	Bovine	Brillault *et al.* 2002 [82] Culot *et al.* 2009 [83]
Conventional medium	Medium used in the conventional culture conditions with low glucose concentrations or without glucose	Human	Slevin *et al.* 2009 [41]
		Mouse	Milner *et al.* 2007 [66]
		Bovine	Dehouck *et al.* 2002 [84] Abbruscato *et al.* 2004 [80] Benchenene *et al.* 2005 [81] Paulson *et al.* 2006 [85]
